# “I Spent Most of Freshers in my Room”—A Qualitative Study of the Social Experiences of University Students on the Autistic Spectrum

**DOI:** 10.1007/s10803-021-05125-2

**Published:** 2021-06-28

**Authors:** Helen Goddard, Anna Cook

**Affiliations:** School of Psychology, University of Surrey, Guildford, GU2 7XH UK

**Keywords:** Autism, University, Education, Disclosure, Young adults, Mental health

## Abstract

Autistic university students face extra challenges in both their academic and social life. Barriers to socialising appear to be less well understood and supported by universities than academic requirements. Semi-structured interviews were conducted with ten autistic university students to explore their social experiences. Questions explored their social experiences, satisfaction with social life, disclosure of ASD to others, and the impact of mental wellbeing on university life. Thematic analysis indicated most participants were unsatisfied with their social lives and experienced mental health issues. Factors exacerbating social isolation included lack of suitable social events, lack of social support and feeling unable to disclose to peers. Factors which reduced social isolation included joining an autism or special interest society and receiving social mentoring.

## Introduction

An increasing number of autistic students are entering higher and further education and completing a degree programme (National Autistic Society, 2016), and therefore the need for universities to address the specific challenges which these students face is more urgent than ever. Recent data show that the number of UK domiciled students who identify as having Autistic Spectrum Disorder (ASD) or Social Communication Disorder is increasing significantly every year, from 6775 in 2014/15 (0.3% of the total student population) to 12,815 in 2018/19 (0.54% of the total student population) (Higher Education Statistics Agency, 2019). Studies have shown that students diagnosed with ASD are likely to face extra challenges in both their academic and social life at university as well as finding it harder to adjust to their new routines and a more independent lifestyle (Anderson et al., 2017; Gurbuz et al., 2018). Gurbuz et al. (2018) compared the social and academic experiences of autistic and non-autistic students enrolled in UK universities and found that autistic students had significantly lower scores in both components (*p* < 0.001) and reported higher levels of loneliness and mental health difficulties than neurotypical students. In addition to experiencing higher rates of depression and anxiety than the general student population, autistic students are also far more likely than average to fail to complete their course and this may lead to poorer prospects in adult life (Jackson et al., 2018).

Most of the literature concerning ASD in educational settings is focused on the experience of school children, and until recently there has been little written about university students despite late adolescence and early adulthood being a developmental period of critical importance. In the last five to ten years however there has been increased interest in the topic (Anderson et al., 2017; Cai & Richdale, 2016; Cox et al., 2017; Gelbar et al., 2015; Gurbuz et al., 2018; Jackson et al., 2018; Van Hees et al., 2015). However, the area of research is still relatively unexplored. The literature includes studies from several countries including the United Kingdom, the United States, Australia, Belgium and the Netherlands and encompasses a variety of concerns including academic performance (Gurbuz et al., 2018; Jackson et al., 2018) discrimination and bullying on campus (Gelbar et al., 2015; DeNigris et al., 2018), mental health issues (Jackson et al., 2018; Van Hees et al., 2015), friendships (Gurbuz et al., 2018; Jackson et al., 2018), sensory issues (Van Hees et al., 2015; Anderson et al., 2017), level of support provided by the university (Anderson et al., 2017; Cai & Richdale, 2016; Cox et al., 2017; Lindsay et al., 2018) and disclosure of ASD (Cai & Richdale, 2016; Cox et al, 2017; Gelbar et al., 2015). Similar topics and concerns are raised in most of the studies suggesting that there is little cross-cultural variation in the countries where this topic has been looked at so far.

The problems resulting from the social aspects of ASD also appear to be less well understood and supported by universities than academic ones, with a study carried out by Cai and Richdale (2016) showing most students they surveyed felt educationally but not socially supported. This is likely to be because many non-academic difficulties indicated by the respondents often require complex and long-term intervention whereas some educational concerns may have more manageable and proven solutions such as additional time in examinations and help from support staff (Anderson et al., 2017). Gelbar et al. (2014) state that much of the evidence supports the idea that social and communication issues are underlying many of the problems experienced by autistic students, even problems that do not appear directly related. This has been supported by others, for instance Jackson et al. (2018) and Anderson et al. (2017) both found that even though most of the self-reported strengths of autistic students can be helpful to academic work, such as detail orientation and strong memory, even autistic students without a learning difficulty may still struggle academically due to difficulty communicating in the classroom, taking part in group work and following instructions that may be unclear.

One area of social interaction consistently highlighted as important is friendships, with the development of friendships being seen by autistic students as an important marker for a successful and enjoyable university experience (Altman, 2014). Gurbuz et al. (2018) used an online questionnaire to survey 26 students diagnosed with ASD in the UK and reported that whilst these students reported a similar motivational level for acquiring friendships to neurotypical students, 72% did not find their relationships with others meaningful, and 66% reported having no friends. This shows that concerning friendships there is a significant gap between desired experience and reality for most autistic students. Whilst it must be noted that the study found that a small minority of these students are content to not have any friendships and prefer to be solitary, most found that a lack of friendships detracted from their university experience and led to loneliness and feelings of isolation (Van Hees et al., 2015).

An aspect of social interaction that is relevant to autistic students is the decision of whether to disclose their condition to peers and members of staff, as others may not be aware unless explicitly told. Studies concerning disability disclosure at university have often focused on disclosure to official support services and the effectiveness of the help they provide (Anderson et al., 2017; Lindsay et al., 2018) rather than disclosure in a social context. In studies where disclosure to peers has been mentioned, it is stated that only a minority of autistic university students choose to disclose to fellow students (Gelbar et al., 2015; Van Hees et al., 2015), and often took a pragmatic approach, disclosing only when circumstances required it (Cox et al., 2017). Students who did disclose to peers were normally very discerning in how they chose to tell others due to past negative experiences, fear of discrimination or labelling (Cai & Richdale, 2016).

The decision of whether and when and to whom to disclose is not straightforward for many autistic students. Studies about disability disclosure have indicated that as well as the negative consequences listed previously, disclosure could have benefits such as reduction of stress as people do not have to constantly hide the challenges they are facing (Van Hees et al., 2015), acceptance from others towards their differences, and it could contribute to the bonding process with friends and increase authenticity in relationships (Blockmans, 2015). Research is therefore needed to explore the tendency of students to disclose their condition to other students (both acquaintances and friends) and to members of staff, the effect that disclosure has had on their relationships, and whether they feel disclosure or non-disclosure has affected their wellbeing.

Overall, the literature reviewed suggests that there are many potential barriers to socialising and friendship at university for students with ASD, for example, not feeling socially supported by the university (Cai & Richdale, 2016; Gubuz et al., 2018), having no friends (Gubuz et al., 2018) and loneliness (Van Hees et al., 2015). It is important therefore to consider the facilitators which may lead to better outcomes. This is an under-explored area compared to the barriers, but studies report benefits from the presence of social support initiatives such as peer mentoring (Thompson et al, 2019), support with the transition from school to university (Lambe et al., 2019) and greater levels of peer education on ASD (Campbell & Barger, 2014).

Various psychological theories attempt to explain why autistic people may find it harder to make friends and why they may perceive friendship differently to others. The most widely known theory is that autistic people have a deficit in ‘theory of mind’, the ability to recognise another’s mental state as distinct from their own, and they therefore find it more difficult to predict and interpret the behaviour of others (Baron-Cohen, 1988). Another recent theory that is currently being investigated is the social motivation hypothesis (Chevallier et al., 2012) which argues that autistic people find social stimuli less rewarding than neurotypical people and for this reason engage less in social situations, preventing the typical development of social skills.

Broader psychological theories may also be useful in analysing social behaviour in autistic and neurotypical people. An example of a relevant theory is Social Identity Theory (SIT) (Tajfel, 1979), which refers to how self-concept is derived from perceived membership of a social group and states that members of an ‘in-group’ will often seek to find negative aspects of an ‘out-group’ in order to enhance their own social status and self-image. According to SIT, autistic people would be perceived as being part of an ‘out-group’ by wider society and therefore more likely to experience prejudice and social rejection.

In light of these factors, and the scarcity of research exploring the lived experiences of autistic students in higher education, in particular their disclosure to peers and effects of mental health factors, the present study aims to investigate the social experiences of autistic students at university. Specifically, the study will aim to identify what participants feel are the facilitators and barriers to their friendships, how satisfied they are with their social life, the reasons for and consequences of disclosure/non-disclosure of ASD to others, and finally, the effect of the impact of the mental wellbeing of participants on their university life.

## Method

### Research Design

This study has a qualitative design, with participants taking part in semi-structured interviews lasting approximately an hour. A qualitative approach was chosen as it allows participants to expand on their views through telling their stories in great depth, helping to build up a detailed picture of complex issues that are to date, under-explored.

### Participants

Participants were required to be a current university student in the UK with an ASD diagnosis and were recruited through purposive sampling; emails were sent to members of several university autism societies and a recruitment message was posted on their Facebook pages. Posters were also put up around the campus of one university and given to the Additional Learning Support department to distribute. Finally, purposive snowball sampling was used, with participants being asked if they could pass details of the study onto others. Participants were entered into a prize draw with the chance to win an Amazon voucher. The first ten students who met the criteria and contacted the researchers expressing willingness to take part were chosen to be interviewed, after this no further interviews took place.

Ten interviews were conducted, ranging from 35 to 79 min in length, and with a mean length of 58 min. The participants ranged in age from 19 to 26, and six were male and four female. Participants came from four universities (five from university A, three from university B, one from university C and one from university D). Five participants had one or more additional neurodevelopmental disorders, these conditions included Dyslexia, Dyspraxia, ADHD, and OCD. Whilst all participants had a diagnosis of ASD, it cannot be said that the sample was homogenous since ASD presents with a wide range of traits and level of severity and therefore cannot be generalized. Participant demographics are shown in Table 1 (all names are pseudonyms).

**Table 1:** Description of the participants according to socio-demographics

Name	University attended	Age	Gender	Ethnic origin	Educational level	Age of diagnosis	Co-morbid diagnoses	Secondary school(s)/sixth form attended
Lucy	A	20	Female	White British	Undergraduate	15	None	Mainstream then special needs
Naomi	A	26	Female	White British	Postgraduate	19	Dyspraxia, ADHD, Dyslexia	Mainstream
Isabel	A	22	Female	Black Caribbean	Undergraduate	20	Dyslexia	Mainstream
Sam	A	21	Male	White British	Undergraduate	12	Dyspraxia, ADHD, OCD	Mainstream with specialist autism unit
Danny	A	19	Male	Mixed: White & Black	Undergraduate	19	None	Mainstream then special needs
Leon	B	21	Male	White British	Undergraduate	3	Dyspraxia, ADHD	Mainstream then special needs
Rosie	B	21	Female	White British	Undergraduate	15	None	Mainstream
Isaac	B	19	Male	White British	Undergraduate	2	Dyspraxia	Mainstream
Joel	C	25	Male	White Other	Postgraduate	24	None	Mainstream
Harry	D	20	Male	White British	Undergraduate	3	None	Special needs

### Procedure

The interview schedule was designed to encourage participants to talk about many different aspects of their social experiences at university. A semi-structured interview format was chosen as this would give participants greater freedom to focus on the topics which are most important to them and allow them to discuss topics unforeseen by the researchers. The initial interview questions were developed by the primary researcher and then reviewed by the second researcher, who is an expert in the field of autism. A pilot interview was carried out with a student who has been self-diagnosed with ASD for several years following extensive research of the diagnostic criteria and is now awaiting a formal assessment on the recommendation of his GP. Following the pilot interview adjustments were subsequently made to the wording and ordering of questions. The interview covered (i) circumstances of their ASD diagnosis, (ii) school experiences (iii) social experiences and friendships at university, (iv) mental health, (v) support received from the university, (vi) disclosure to other students and teaching staff and (vii) perception of the awareness of other students of ASD. All interviews were conducted in a private study room at the university that the participant attended. The full interview protocol can be found in Appendix A. Interviews were recorded and transcribed with the participant’s permission. This study was granted ethical approval by the University Ethics Committee (reference FER-1819-043). Each participant was provided with an information sheet that stated that their participation was voluntary and that they could withdraw from the study at any time without explanation.

### Data Analysis

Thematic analysis was chosen as the method of data analysis as it has the potential to provide a rich and detailed yet complex account of data, and is also a useful method for highlighting similarities and differences between participants and generating unanticipated insights (Braun & Clarke, 2006; King, 2004). A rigorous thematic analysis can produce trustworthy and insightful findings (Braun & Clarke, 2006) and produce a clear and organized final report (King, 2004). Similar studies on autistic university students have used thematic analysis to good effect, including Gurbuz et al. (2018) and Cai and Richdale (2016).

The analysis was carried out in the following stages, closely following the model outlined by Braun and Clarke (2006). First, the researchers identified repeated patterns of meaning across the data set, from which themes were then identified. An inductive approach was taken, whereby the themes are primarily derived from the data rather than being theoretically driven. Analytic stages included familiarisation with the data by both authors through detailed reading of interview transcripts, working systematically through the data set to produce initial codes. The coding was done by the first author to allow for continuity between interviewing, coding and analysis across participants. Each interview was coded line by line and tentative links were posited between participants. Once regularities were identified in the data between participants, the codes were then sorted into potential themes and sub-themes in collaboration with the second author, the refinement of the selection of themes, and finally the analysis of themes and sub-themes to ensure that they reflected coded extracts and consideration of how they fit into a broader context. Agreement was achieved between researchers as an iterative process. The research was evaluated using elements of the evaluative criteria by Elliott et al. (1999). These included the researchers owning their perspective through recognising their own values and assumptions and the role these play in their understanding; situating the sample; grounding in examples that allow appraisal of the fit between the data and the authors’ understanding of them; checking of the credibility of themes by the second author, who had extensive professional and personal experience of working with autistic young people; and resonating with readers by presenting the material in such a way that readers judge it to clarify their appreciation of the subject matter.

A sample of 10 was used, in accordance with guidelines for thematic analysis, suggesting that for a small project using interviews, 6–10 participants are recommended (Clarke & Braun 2013). Theoretical saturation (Glaser & Strauss, 1967) can be achieved after as few as six interviews (Isman et al., 2013). However, Francis et al. (2010) suggested that ten interviews were necessary for saturation to occur, meaning that the addition of more participants would not lead to the introduction of new themes, and therefore provide a sound sample. A theme was considered saturated when it showed a level of prevalence across the data set or when it was given considerable space within one or more data items, in line with Clarke and Braun (2013).

### Role of the Researchers

The first author’s role was to select participants, devise and conduct the interviews and analyse the transcripts. A hermeneutical approach was used (Gadamer, 2004), meaning that the researcher must be aware of their own pre-understanding (existing knowledge, values, and experience) of the topic of interest when they interpret the accounts of participants’ experiences. Participants’ experiences are brought together with the researcher’s to produce a new understanding of the area of interest. In this study, the primary researcher (first author) was a neurotypical female graduate student, whose pre-understanding consisted of theoretical knowledge and personal experience of working with children on the autistic spectrum. It is acknowledged that due to not being autistic, the interviewer’s characteristics may have influenced the interviewees’ responses. However, being of a similar age group and socio-economic background to the majority of participants brought a level of empathy to the development of questions and to the interview process, and she was able to pick up on finer nuances in participant responses. The second researcher (second author) was a neurotypical female teaching fellow in developmental psychology with considerable expertise in the field of autism.

## Results

**Fig. 1:**
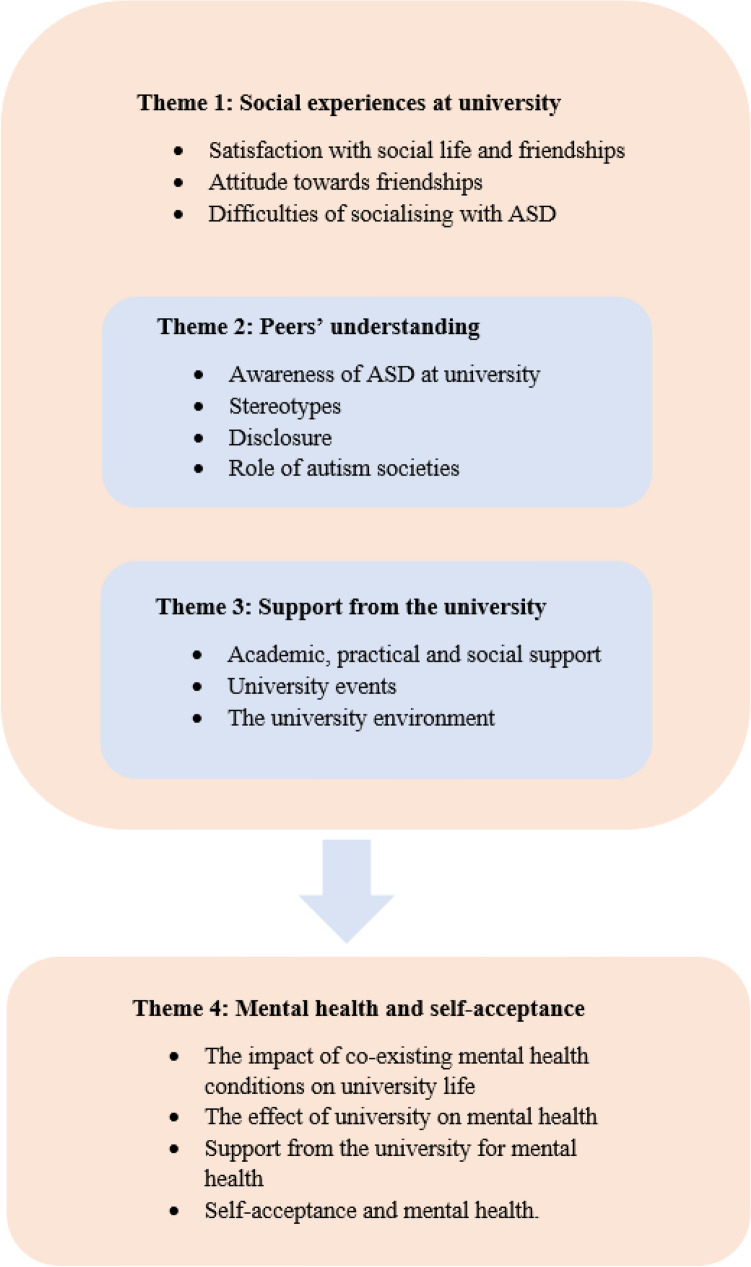
An overview of themes and sub-themes

The data collected were divided into the following themes: (1) social experiences at university, (2) peers’ understanding, (3) evaluation of university support and (4) mental health and self-acceptance. Each theme was divided into several subthemes, as shown in Fig. 1.

### Theme 1: Social Experiences at University

#### Satisfaction with Social Life and Friendship

Just over half of participants reported dissatisfaction with their social life during their undergraduate degree, ranging from mild to severe. Most participants reported having mainly acquaintances and a few individual friendships, and several described struggling to make any friends at all, but it is important to note that there was not always a direct relationship between the number of friends and acquaintances and satisfaction levels. Sam reported that his biggest concerns about university life were the difficulties he faced socialising with peers. “That’s probably been the most difficult thing for me at university. I don’t struggle with exam preparation, doing essays, stuff like that. What I struggle with most is things like going to the pub with friends after class.”

Naomi also described struggling with socialising during her undergraduate degree. “I was constantly on the outside, more like a satellite on friendship groups, so there was this pervasive feeling of loneliness.” In contrast, Rosie reported becoming more satisfied with her social life since finding a small group of friends she trusted and had become close to. “I’ve been quite lucky to find a nice little friendship group who are all quite quiet and have anxiety issues as well and social issues, but it’s quite hard to integrate with other people on the course.”

Some participants reported being satisfied with their social lives, for instance Isaac described having at least five friends on his course and being surprised at how well he adapted to university life. However, whilst two of the four participants who stated they were satisfied reported a varied and active social life, the other two stated that they had only a few friends and acquaintances and socialised less than they felt was typical. Lucy reported the following: “I think a lot of people wouldn’t be [satisfied]. I think a lot of people want more but I’m quite happy with a more limited social life.”

#### Attitudes Towards Friendship

Participants differed on how much importance they placed on having friends at university although most stated that it was important. Rosie explained why having friends was so crucial to her: “I need a safety net of people when things get tough, I need to be able to talk to people and do activities with them regularly otherwise I get really isolated.” Naomi also agreed on the importance of friendship and contrasted her feelings to how she felt autistic people are perceived by others to feel about friendships.

There is this stereotype with autism that people have where they think that autistic people don’t want friends. No one doesn’t want friends, we’re not soul-less, and we do. It’s just harder and it’s only really when you become an adult that you have the opportunity to mingle with people who are like you. (Naomi)

A common theme was that the quality rather than quantity of friendships was most important, and respondents tended to mention looking for personal qualities such as authenticity, trustworthiness and non-judgementalism in a potential friend. Several participants said that they would rather have no friends than friendships that they felt were not of high quality. Isabel summed her views on the importance of friendship in the following way:

The importance [of friendship] in my opinion is based on the quality of the friendships. If someone wanted to be friends with me, and that friendship was going to be good, then I would find that important. But just the idea that having friends, just any friends, is just not important. I’d rather just be on my own than have a friendship that isn’t great. (Isabel).

#### Difficulties of Socialising with ASD

The majority of participants reported that social and communication difficulties were a barrier to making friends and socialising at university. The nature and severity of these difficulties varied greatly. Difficulties that participants attributed to having ASD included anxiety whilst socialising, overthinking social situations, misjudging social cues, difficulty making conversation, difficulty articulating thoughts/formulating sentences, difficulty making eye contact, finding socialising mentally draining, talking too much and talking over others. Sam described how he feels having autism makes it more difficult for him to pick up on social cues:

I miss the social cue about when it's my turn to start speaking and I'll start talking over them. I feel like that harms my friendships because people get annoyed with it, they think “oh god he always talks over people, he never listens to people. (Sam)

Several participants reported sometimes feeling confusion whilst navigating social situations. Naomi described how she feels not being aware of unspoken social rules made it more difficult for her to maintain friendships during her undergraduate course. “I couldn’t keep friendships. I would try really hard, but I couldn’t really understand what was happening. There are so many unspoken social rules that I couldn’t keep up with.” It was common for participants to report that they felt they over-analysed social situations and that this could be mentally draining. Leon explained how he often rehearsed scenarios in his head both before and during the interaction.

I tend to measure every word I speak just in case I’m giving anything away, trying to rehearse every scenario if I’m going to be socialising, and then it looks more natural hopefully, It’s kind of impossible though. So socialising isn’t really something I manage to do successfully. (Leon)

### Theme 2: Peers’ Understanding

#### Awareness of ASD at University

Most participants felt that although other students were familiar with the term autism, they had little knowledge of the condition unless they had direct involvement with someone with it. Lucy explained how lack of awareness meant she was often wary of telling other students she had autism:

I don’t think they are [aware] at all. Most people know it’s to do with social interaction, but it’s often used as an insult. I hear it used as an insult sometimes, and I just think they don’t know anything about it. (Lucy)

The vast majority of participants felt that awareness of autism at university could be improved, however most were not sure how this should be achieved. Strategies already in place which were seen as positive included fundraising events and the creation of societies for autistic students. Whilst the creation of an awareness day or week with informative events was suggested by several participants, some were concerned that efforts at raising awareness could feel forced and patronising and would not be willing to be involved themselves due to the reaction they might get from others.

#### Stereotypes

There was a strong feeling that other students were likely to base their knowledge on stereotypes that portray autism negatively. Stereotypes mentioned included not being able to feel or express emotion, inability to empathise, not wanting to have friends, being rude, having savant abilities, having niche interests, and being child-like. These stereotypes were sometimes reported as being spread on social media through derogatory memes. It was also felt that there was not enough appreciation of the fact that autism traits exist on a spectrum and manifest in many different combinations. Leon explained how he felt that the media portrayal of autistic people contributed to the perpetuation of stereotypes. “There’s literally every kind of insensitive movie where they will read the Wikipedia page and just give the character every symptom. Unrealistic.”

Isabel considered media portrayal to encourage the idea that rather than being on a spectrum, autistic people are likely to fall into one of two groups: non-verbal with severe learning disabilities, or extremely capable academically but socially inept.

If they’ve got the Sheldon Cooper stereotype in their mind, they just think you're probably rude and incredibly smart. Some people just really have no idea what ASD is. They may not even realise that autistic people can go to university. (Isabel).

Naomi described how she feels the prevalence of stereotyping has led to her choosing not to disclose her autism to others with who she interacts on a professional level. “People associate it with being unstable, asexual, and I think they kind of infantilise you, so you become this child-like figure. Even if you’re competent academically, you’re still incompetent because you must be incompetent socially” (Naomi). However, a few participants found that peers who they disclosed to had been open to understanding them as individuals rather than relying on stereotypes. Harry described how other students’ responses had been more positive than he was expecting.

I make the misjudgement of assuming that some people know less or think more negatively about it than they actually do. Most of the time people are more like, “whatever, that’s a thing, I’m not going to treat you any differently for this.” (Harry)

#### Disclosure

All participants reported having disclosed their ASD to the Additional Learning Support department at their university, and six had told at least one member of the teaching staff. Rates of disclosure to other students without ASD ranged from having told no one (2), a few close friends (4), 5–10 friends and acquaintances (2) and “everybody knowing” (2). Several participants stated that they felt disclosing to friends was positive for their emotional wellbeing and most felt the effect on friendships was positive or neutral. Previous experiences of disclosure, particularly at school, influenced many participants in their decision. Leon was bullied after his peers at his school found out about his diagnosis and now avoids socialising at university as he feels paranoid about others finding out. “My biggest concern about life in general is people finding out about my diagnosis and then behaving irrationally because of that. That’s happened too many times for me to not worry about it.”

Rosie also experienced bullying as a result of disclosing ASD, and so is now selective about who she tells at university.

In the past, I’ve opened up or been weird and told someone that I have ASD, and they’ve bullied me about it. Now, I’ve analysed people and tried to make sure they’re going to be the kind of person that doesn’t judge me. (Rosie)

It was common for participants to report only telling friends that they felt met certain criteria. These included being knowledgeable about ASD, having a close friend/family member who has ASD and personal qualities such as being non-judgemental and trustworthy. Others reported being less concerned with others reacting negatively but that they would only mention it if relevant to the conversation. Finally, several participants felt that their feelings towards disclosure had become more positive since they first started university and that over the years they had become more relaxed about telling others. Naomi, a postgraduate student, described how she has now chosen to make most people she knows aware that she has autism:

At first, I wouldn’t really tell anyone, but I think it’s like a process. Back in 2016 I did a talk, and it was all about how my life is as an autistic person and, at that point, lots of people found out and since then I’m just like, “yes, I’m doing this because I’m autistic, this is how I am”. So now, I don’t particularly hide it, but it’s taken time to get to that point. (Naomi)

#### Role of Autism Societies

Seven participants were members of a university autism society. Those who attended regularly felt that meeting others with autism was a beneficial experience as it allowed them to develop friendships and share experiences with students they could relate to. Isaac attends the autism society at his university every week and explained why he found the sessions to be so beneficial. “Up until that point I hadn’t really experienced being around others with autism, so having a roomful of people that I could understand to and relate to in that way was really helpful.”

Whilst some participants preferred to keep their participation private from other students, several felt it had acted as a catalyst for them to disclose to others, for example Isabel explained how taking part in promotional activities at Freshers’ Fair meant that she decided to tell friends who were not previously aware. Most participants who attended such societies found it to be beneficial, although it was mentioned that attendance was generally low (5–15 people) at meetings in all three societies. This meant that there could be a lack of diversity, for example in one society almost all the regular attendees were male and engaged in similar interests, and this was off-putting to a female participant who stopped going as a result. Several participants who chose not to attend stated they preferred going to mainstream societies that catered to their interests and enjoyed socialising in this environment, and so felt that specifically spending time with other autistic students was not necessary.

### Theme 3: Evaluation of University Support

#### Social Support

Participants at one university reported they benefitted from being able to move into their accommodation several days early and take part in an induction programme where they could meet other autistic students. Five participants in total across the universities reported seeing a social mentor each week and each had a positive evaluation of the effect it had on their wellbeing and on helping them navigate social situations. Rosie described how essential she found both the study skills and social mentoring. “I think that’s the main thing that has really got me through this year. If I didn’t have these two people to help me make a structure I would just collapse.”

Harry reported that at his university autistic students could take part in a scheme where other students, who have volunteered as a social mentor, accompany them to events. Whilst he did not feel he needed a student mentor himself he knew others who had found it extremely helpful. Sam felt that a scheme such as this should be available at all universities, and stated: “I feel like if I had a person my age that was also studying their degree it would help me with building friendships and make me feel more confident with going out.”

#### University Events

Only three participants reported participating in Freshers’ Week, and of those two chose not to continue because of events being too noisy and feeling overwhelmed. Lucy reported that she did not enjoy any events she attended and described how the crowds made her feel anxious. “[it was] Stressful. We had Freshers’ fair, and I tried to go to that, but it was so packed that I just had to leave straight away.”

Isabel reported being disappointed with her experience overall and felt isolated due to being uncomfortable attending events where there was heavy drinking.

I just heard everyone saying it’s the best experience of your life. So I got here, and I just assumed that was going to be more non-drinking events than there were. And there weren't, so I spent most of Freshers’ in my room. (Isabel)

Whilst it was acknowledged that there are many societies that run a variety of events, most participants felt that going out socialising with housemates and course mates often involved conditions that they felt uncomfortable in such as crowded spaces with loud music. Most were content in their decision to avoid such events, however Sam felt it was important to him to take part and have a more typical university experience.

I don't necessarily want segregated activities, I want to be included on what's already there, I just want help getting there and I feel like student mentors is what the university needs to pursue, and buddy schemes. What I'd like is a mentor who perhaps says, “I'm going clubbing on this night do you want to come, and I'll stay with you and make sure you feel comfortable.” (Sam)

#### University Environment

Most participants felt that they were often adversely affected by the university environment. Issues raised included lack of quiet spaces, narrow corridors and bright lights and repetitive noises in lecture halls. Naomi reported she stopped attending a certain lecture each week due to the conditions inside: “There was one [lecture theatre] in particular that had bright fluorescent lighting, no windows, you were squished up with a lot of people. It was always full and it used to give me panic attacks.”

Whilst Joel reported feeling relatively comfortable in lecture halls, he found moving to and from lectures to be more challenging:

The in-between movements are probably the most stressful bit, in heavily populated universities such as this there is lots of noise and chaos. That’s one of the moments when you have to figure out how you are going to avoid the stimuli to cope better.” (Joel)

Aspects of the environment that were praised included small individual study rooms, single space work booths in the library and new buildings with a spacious environment. Several participants suggested that it should be mandatory for recordings of lectures to be available online as there were some days where they were unable to cope in lecture theatres.

### Theme 4: Mental Health and Self-acceptance

#### The Impact of Co-existing Mental Health Conditions on University Life

Most participants reported that they had been diagnosed with at least one mental health condition and several participants had two or three comorbid conditions (specific conditions reported in Table 1). Lucy, who was diagnosed with Generalised Anxiety Disorder when she was 15, described how when her anxiety levels are high she feels sick and feels pain in her body, and that this has affected her ability to socialise at university: “In the first couple of weeks when people were trying to make friends, I completely shut down and refused to talk to anyone because of it. By that point they’d all made friends.”

Four participants had been diagnosed with depression, and several more said that they experience mild symptoms. Joel described how he has struggled to find an effective treatment for his severe ongoing depression, and that this greatly impacts his life at university and elsewhere:

I’ve been dealing with depression for a long period of time now, going on a decade. There’s been lots of ins and outs with depression, lots of moments where you don’t want to do anything, lots of moments where you contemplate suicide.” (Joel)

Several participants felt that their current mental health issues had been caused or exacerbated by past incidences of bullying. Whilst none of the participants reported being bullied at university, half reported being bullied at school, and of these, four reported it having directly affected their mental health*.* Rosie explained how she feels having ASD affects her mental health and how past incidences of bullying are still affecting her today:

It causes me to second guess everything, over-analyse everything, and to have a lot of intrusive thoughts and obsessive spiral of thoughts. So, it’s pretty disorientating and you can swing from one mood to a totally different mood in an hour or even less than that. I have triggers from the past, for example if someone has bullied me then if people say something similar now it will trigger the same thoughts. (Rosie)

#### Effect of Attending University on Mental Health

It was reported that attending university brought up various challenges which could have an impact on mental health including the strong emphasis on socialising, lack of a structured routine, difficulty adjusting to independent living, distance from friends and family, feeling isolated and struggling to keep up with the workload. Whilst these are issues that can affect all students, it was felt that it may be harder for autistic students to adjust to dealing with these situations. Naomi reported that keeping up with daily chores such as cooking and laundry was the most challenging aspect of living independently for her, whilst Rosie felt that the lack of routine, particularly during exam periods, was making her depressive symptoms worse. Rosie also reported she had become concerned about her alcohol consumption, which she felt had become an unhealthy coping mechanism. “I went down quite a dark place, especially in the winter with depression. I generally drank in social situations to get through them and be a lot less aware of myself, and then that leached into my everyday life.”

Feeling socially isolated was also mentioned as a reason for depression and anxiety and this could worsen due to living apart from family for the first time. However, most participants also mentioned how attending university has affected them positively in some way, including having more freedom, having their own space, becoming more independent, more choice in who to socialise with, and being able to pursue their interests.

#### Support from University for Mental Health

Half of the participants reported having accessed support for their mental health from university services, in most cases in addition to ongoing treatment elsewhere. Of these, most felt that seeking help with university services had been helpful. Harry, for example, had a positive experience of receiving counselling at university and described his confidence increasing as a result, although reported having to wait several months for an appointment. Joel also found counselling beneficial overall, although he felt having ASD could make it harder to receive effective treatment:

It’s been helpful, every now and again. Another hard part when you’re on the spectrum is that when you go to counselling it’s hard for you to be able to convey yourself and be interpreted properly to then receive proper advice.” (Joel)

One participant expressed dissatisfaction with her experience as she felt that it was harder for autistic students to access help at university due to their needs being seen as too complex. “They were like ‘we can’t cope with you.’ I know that’s an experience shared by a lot of autistic people, where the University mental health services won’t work with them because you fall into the ‘too complicated’ bracket.”

#### Self-acceptance and Mental Health

Several participants gave their thoughts on self-acceptance of ASD and ASD related traits. Isabel felt that she had been through a process of self-acceptance after spending years being isolated socially at school and now feels that the good points of having ASD outweigh the bad: “Autism isn't something wrong with you, it’s just being different. Other people shouldn't say I'm so sorry to hear you've got Autism. It's just having your brain wired a bit differently and that's not a bad thing.”

Many participants felt it was beneficial to them to note possible ASD-related traits which had a positive effect on their lives. These varied and included the ability to focus intensely for long periods, being analytical, having a good memory, the ability to see things differently to others and personal qualities such as authenticity, reliability and staying true to their values. Harry explained how having ASD could bring benefits to others around him as well as himself: “Because I have a different way of thinking I could bring a diverse viewpoint to a workgroup to solve a solution. You need people who can think in different ways.”

Rosie felt that one of the hardest things about having ASD is that the world is centred around the needs of neurotypical people, but that she has now reached a point where she did not feel the need to try and change herself for others:

ASD has made it easier for me to be myself in front of people because I don’t really have a choice. If I tried to be normal all the time, it would just kill me, it would make me absolutely crazy. So, it’s quite nice that I have to embrace who I am. (Rosie)

Leon explained how he found it difficult to come to terms with having a condition that differentiated him from others, but now actively works on focusing on the positives of ASD. “I fought very hard to make it a positive thing for me. It was really quite difficult, so I just tried to see it as a superpower.”

Most participants felt relief upon receiving their diagnosis however several also described this as being mixed with feelings of fear, confusion and doubt over their symptoms. Naomi explained how despite many around her initially having a negative reaction to her having autism, she has come to fully accept and embrace having ASD. She feels however that this process was hindered by the negative judgement of autism being so commonplace:

My initial reaction was this isn’t a bad thing but then lots of people around me were telling me “it’s a bad thing”. But once I came out of that slump, it’s only been a positive thing. It allows me to understand myself, it allows me to find people who are similar to me and be like “I’m actually normal” but it’s just a different type of normal. (Naomi)

## Discussion

This study aimed to explore the social experiences of university students diagnosed with ASD, focusing specifically on satisfaction with social life, the facilitators and barriers to a positive social experience, the reasons for and consequences of disclosure and non-disclosure of ASD to others, and finally, the impact of mental wellbeing on participants’ university life. The research questions asked: what are the experiences of friendship and socialising of autistic university students, and what is the impact of mental health and wellbeing on university life? Overall, the results suggested that although there were several exceptions, most participants had experienced social difficulties at university and that most were not satisfied with their social life, and that this may be associated with lower mental wellbeing.

Most participants stated that they were not satisfied with their social life, a finding which is largely supported by previous studies on autistic university students (Gurbuz et al., 2018; Jackson et al., 2018; Van Hees et al., 2015). The results suggested that whilst having a busy social life may not be a high priority for most autistic students, most do desire friendships and wish to form meaningful connections with others, as found in previous studies (Gurbuz et al., 2018; Van Hees et al., 2015). There were however several participants who felt more positively about their social life who reported having a small group of supportive friends with similar interests and personality traits, and it seems likely that finding such a friendship group increases feelings of self-acceptance and self-esteem, supporting previous studies exploring perceptions of self-esteem and friendship in autism (Bauminger et al., 2004).

Specific barriers and facilitators to a positive social experience at university were identified in the results. One of the major barriers mentioned by participants was the detrimental effect that certain inherent traits of ASD, such as communication and sensory sensitivities, had on their ability to socialise at university and make new friends. This was compounded by the anxiety some participants felt about socialising due to having been bullied or socially excluded during their school years, further reducing confidence in socialising at university. Barriers to socialising which were more specific to the university environment included being unable to attend social events due to a loud or crowded environment, supporting previous studies finding that sensory issues increased the difficulties attending social events (Van Hees et al., 2015) and encountering a lack of understanding or knowledge from peers concerning ASD.

Facilitators for a more positive social experience at university included having adequate support from the university (including social mentoring), having attended a supportive school, spending time with other autistic or non-neurotypical students, being involved in a society for a specific interest, and having at least one high-quality friendship with a student who is accepting and aware of ASD. Similar protective factors for a positive social life and mental health were identified in a study of autistic adults (Cage et al., 2018), which suggested that the presence of one of these factors could disrupt the negative feedback cycle of risk factors described above.

Peers’ understanding was perceived to influence participants’ social experiences at university. Whilst several previous studies have briefly mentioned disclosure to peers in a university setting (Cai & Richdale, 2016; Gelbar et al., 2015; Van Hees et al., 2015), this is the first time it has been examined in greater detail. The results indicated that most participants were very selective in who they told due to negative past experiences, and/or the stereotypes they believed others held about ASD. In particular, participants who had previously experienced bullying at school were less likely to disclose to their peers at university. There was also a trend towards younger undergraduate students being more reluctant to disclose to peers, whilst the two older postgraduate participants stated that they were now open with almost all their peers about having ASD. Overall, when disclosure to peers did take place it was generally seen as a positive or neutral experience, and beneficial to the mental wellbeing of the participants, as previously reported within school settings (Ochs et al., 2001).

Many participants felt that other students were likely to base their knowledge on stereotypes that portray autism negatively. Stereotypes cause an overestimation of similarities among people with a particular category (Tajfel, 1963) leading to a generalisation of a group as a whole, particularly when we are unfamiliar with the category (Quattrone & Jones, 1980). Stereotypes of autism therefore may arise due to unfamiliarity and the inability of neurotypical individuals to fit various autistic characteristics into pre-existing schemas. The resulting stereotypes are limiting and destructive (Botha et al., 2020), leading many autistic people to attempt to conceal their autistic characteristics (Hull et al., 2017).

Participants frequently referred to the detrimental effect that stereotyping, and fear of being stereotyped, has had on their social lives and mental health, and is the main reason given for non-disclosure of ASD to peers. Several participants mentioned that they had seen content shared by other students on social media or private messaging groups which stereotyped autism in a negative light, highlighting that although active bullying at university may be relatively rare, social exclusion through the perpetuation of stereotypes is likely to be occurring.

Whilst there were a few exceptions, most participants were unsatisfied with their social life and described often facing feelings of loneliness and isolation during their time at university. Several participants linked this directly to a decline in their mental wellbeing, stating that loneliness and isolation had led to the worsening of depression and/or anxiety. However, for a few participants, mental wellbeing remained unchanged, and for some it improved after starting university due to factors such as no longer experiencing bullying, having the ability to seek out those with similar interests for the first time and being able to access counselling and mentoring. Overall, whilst it seems there is likely to be a link between mental wellbeing and quality of social experience at university, it is difficult to ascertain the strength of the link and whether it may indicate primarily correlation or causation.

It is important to note that when assessing mental health outcomes that around 70% of autistic people in the UK have a mental health condition, as opposed to around 25% of the general population (MQ Mental Health Research, 2021) and therefore students with ASD are at a higher risk of poor mental health than neurotypical students. Experiences prior to starting university are also likely to have played a large role in mental health outcomes, for instance almost all participants who reported being previously bullied at school reported a current mental health condition. In addition to this, every participant will have experienced ASD related symptoms, such as communication difficulties and sensory sensitivity, to a different degree, and this will have affected their ability to both socialise and reach their academic goals at university with varying levels.

### Psychological Theory

This study has implications for psychological theory. As formerly mentioned, reduced theory of mind—the ability to recognise others’ mental states as distinct from one’s own—has been used to explain the social deficits associated with autism (Baron-Cohen, 1988). This theory helps to explain the findings to some extent, in that participants reported difficulties making conversation and misjudging social cues as barriers to making friends and socialising. Conversely, the social motivation hypothesis (Chevallier et al., 2012), which argues that autistic people engage less in social situations due to finding social stimuli less rewarding, was not widely supported by these findings, since most participants desired friendships and meaningful connections with supportive others. Neither theory fully accounts for the range of challenges faced by the participants, for example difficult attending social events was also reported to stem from sensory issues caused by noise and overcrowding.

Social Identity Theory can be used to explain some of the participants’ views regarding disclosure and attitude of peers as well as how strongly they identify with and accept their ASD diagnosis. A recent study on the subject of disclosure of disability and LGBTQ identity at university (Miller et al., 2018) uses SIT to explain some of the reservations that students may have about disclosure, as they would be identifying themselves as part of an ‘outgroup’ which is likely to change the perception of others towards them and possibly lead to increased social rejection. Whilst Miller et al.’s study was not about ASD directly it can be applied to autism in that disclosure could lead to a loss of ‘in-group’ status. This is supported by the results of the present study as most participants, and in particular those who previously experienced bullying or social isolation, indicated that they are very selective about which other students they tell due to concerns over stereotyping and being treated negatively by others.

Conversely, SIT may also be relevant to the decision to join an autism society, as in this context the individual is joining an in-group in which having an ASD diagnosis is a positive factor that enables group membership and increases the social connection of the individual. This has been largely supported by the results of this study, as most participants who joined an autism society reported that it had been a beneficial social experience and increased their feeling of belonging. It is possible that being connected with others on the autistic spectrum may also positively influence the level of self-acceptance, and in turn ultimately benefit their mental health. SIT has been used to explain how many individuals with disabilities increase self-esteem by employing both individual and collective strategies which minimize stigmatized attributes and positively redefine stigmatized traits (Nario-Redmond et al., 2013). This may explain why in the present study some participants described being motivated to make their autism an important and positive part of their identity, and why this desire may be even stronger if they have previously faced many difficulties in life due to being autistic.

### Implications for Universities

These findings have important implications for improving the support universities provide for autistic students. There is clearly a need for further social support as this is a key area of difficulty for those on the autistic spectrum, and as fundamental to wellbeing at university as receiving support for their academic needs. All students who had mentoring sessions found this very beneficial, and several described it as crucial for their survival at university. However, only one university had a scheme whereby other students could volunteer to be a social mentor to autistic students; this could be an effective and direct way to help autistic students make friends and feel less isolated. Additionally, it is important that staff working in mental health services at all universities are knowledgeable about ASD and the particular challenges that autistic students may face.

There were several other ways in which the present study indicated the university experience of autistic students could be improved. To reduce isolation, particularly during Fresher’s week, there should be enough non-alcohol focused events in environments that are not too noisy or crowded and that these events are promoted sufficiently. The presence of an autism society was also beneficial in decreasing isolation. A further factor causing isolation was lack of awareness of ASD amongst other students and perpetuation of stereotypes which left students with ASD feeling isolated. Whilst more research is required to find the best methods of raising awareness, universities should do more to educate neurotypical students and challenge stereotypes, aiming towards a more inclusive climate that embraces diversity. There is evidence that strategies such as training staff, adapting environments and teaching students about the advantages of neurodiversity have been successful in some mainstream schools in raising awareness and understanding of autism (Campbell & Barger, 2014; Frederickson et al., 2010; Wainscot et al., 2008), and so universities could improve or add to their existing approach with similar strategies. Peer education efforts should include an explanation about autism, highlight similarities between students with ASD and peers, and make direct suggestions for how to interact with a student with ASD (Campbell & Barger, 2014).

Finally, it was found that the university environment was not ideal for autistic students due to overcrowding (especially in corridors), noise levels and sensory stimuli such as bright lights. There are clearly financial barriers to addressing these issues but in the future accessibility of buildings for those with less ‘visible’ conditions such as ASD should be considered. In the meantime, adaptations should be made where possible (for example light brightness in lecture theatres), and lectures recorded and made available online.

### Limitations

There are limitations in the present study to be considered. Firstly, the presence of self-selection bias as there may have been students who might have liked to take part but found the idea of a spoken interview about their personal life too uncomfortable. This could possibly be lessened in future studies by having multiple modes of data collection, for instance online questionnaires, although this may lessen the quality of the data. Secondly, as most participants attended autism societies the study may be more biased towards students who have chosen to disclose their ASD to others and who have a support network in place. This is hard to avoid due to difficulty accessing autistic students who are not in a society but could possibly be reduced by advertising more heavily around campus and social media.

### Conclusion

To conclude, the results of this study provide a rich understanding of the subjective experiences of the social life of university students with ASD. Whilst a few participants reported socialising regularly, most were unsatisfied with their social life, several described having no close friends or no friends at all, leading to feelings of isolation and most suffered mental health issues. Risk factors for feeling socially isolated were inter-related and included both factors directly related to ASD, such as social difficulties and being limited in socialising by sensory differences, as well as external factors at the university such as lack of suitable social events and peers who have little understanding of ASD. These risk factors could however sometimes be mediated by protective factors, such as finding friends with similar qualities through joining an autism society or special interest society, receiving social mentoring on a regular basis or disclosing to peers and finding the reaction is positive or neutral. Either through these protective factors, or other self-protective mechanisms, several participants reported becoming increasingly accepting of their ASD diagnosis during their time at university and turning it into a positive part of their identity, experiencing improved mental wellbeing as a result.

## A: Interview Schedule

My research explores the social experiences of autistic students. I’m going to ask you some questions and ask that you be as honest and open as you can. If you don’t understand a question, please ask me to clarify.

I’d like to start off by getting to know a little bit more about your life as a student.

- How long have you been studying at this University?
- What were your reasons for choosing to come to this University?
- Do you live on campus?
- Who do you live with?

I would like to find out a bit more about your ASD diagnosis.

- Can you tell me a bit more about when you were diagnosed and how that happened?
- How did you feel when you got the diagnosis?

Can you tell me a bit about your experiences at school?

- Can you tell me about your friendships and social life at school?

What do you feel are the positive and negative aspects of having ASD?

Next, I’d just like to find out a bit more about your friendships at university.

- Would you say you're satisfied with your friendships and your social life?
- How important do you feel it is to have friends at university?
- What would you say constitutes a friend in your view?
- What's the most important aspect our friendship for you?
- Can you give me a brief description of your friendships and acquaintances at university?
- Would you say you've got a group of friends, individual friendships, or acquaintances?
- Can you think of a specific person and how often you might meet up with them and what activities (if any) you might do with them?
- How do you feel having ASD affects your friendship and social life?
- Can you describe your friendships and acquaintances outside of university?

The next section is about how you found the transition from school to university.

- What was it like for you socially when you started university?
- How did you find the first few weeks of starting University, were you involved in Freshers Week?
- What are your biggest concerns about University life?

Next, I have a few questions relating to mental health.

- Have you been diagnosed with anxiety, depression or any other mental health conditions either now or in the past?
- If you have not been diagnosed, do you feel like you have experienced one of these conditions, either now or in the past?
- Do you feel that going to university has affected your mental health positively or negatively or not at all?
- Have you had support from the university or elsewhere for any mental health condition?
- Do you feel the help that you received from the university was adequate?

I’d like to ask you some questions about being a member of an autism society (if applicable).

- How long have you been a member?
- How often would you say you go?
- What kind of events do you do there?
- Can you just describe some of your friendships there?
- How many people are there in the society would you say?
- And how often does the society meet up?
- Do you see any of those people outside of meetups?
- What are the benefits of being a member?

Next, I would like to ask some questions about who you decide to tell about having autism.

- Have you disclosed your ASD to the additional learning support?
- What kind of support do you get from them?
- Do you have any mentors? If so, can you describe what you do with them, how often you see them, have you found it helpful.
- Have you told your personal tutor or any of the teaching staff? If yes, was this helpful?
- How many of your friends and acquaintances outside of the autism society have you told that you have autism? Could give a rough number?
- Do you feel that telling others you have autism affects your friendships with them positively negatively or not at all?
- Has telling others affected your mental health or emotional wellbeing?

The next few questions are about how you feel others perceive ASD.

- How aware do you feel other students are about autism?
- What do you think is the general perception of people with autism, and stereotypes that other people might have?
- How do you think awareness could be improved at University?
- Do these stereotypes affect your decision of whether to tell people?

Finally, I'd like to ask you about improvements that you feel could be made by the university.

- Do you feel that socialising at university could be improved for people on the autistic spectrum?
- What kind of social events do you enjoy going to? Do you feel that they are on offer?
- How do you feel about spending time in the spaces at university like the library, the lecture theatre, the restaurant, the cafes? How could this be improved?
